# Volume-replacement ratio for crystalloids and colloids during bleeding and resuscitation: an animal experiment

**DOI:** 10.1186/s40635-017-0165-y

**Published:** 2017-12-20

**Authors:** Ildikó László, Gábor Demeter, Nándor Öveges, Dániel Érces, József Kaszaki, Krisztián Tánczos, Zsolt Molnár

**Affiliations:** 1 Faculty of Medicine; Institute of Surgical Research, University of Szeged, 6. Szőkefalvi-Nagy Béla st, Szeged, 6720 Hungary; 2 Faculty of Medicine; Department of Anaesthesiology and Intensive Therapy, University of Szeged, 6. Semmelweis st, Szeged, 6725 Hungary

**Keywords:** Colloid, Crystalloid, Volume-replacement ratio, Glycocalyx

## Abstract

**Background:**

Fluid resuscitation remains a cornerstone in the management of acute bleeding. According to Starling's “Three-compartment model”, four-times more crystalloids have the same volume effect as colloids. However, this volume-replacement ratio remains a controversial issue as it may be affected by the degradation of the endothelial glycocalyx layer, a situation often found in the critically ill. Our aim was to compare colloid and crystalloid based fluid resuscitation during an experimental stroke volume index (SVI) guided hemorrhage and resuscitation animal model.

**Methods:**

Anesthetized and mechanically ventilated pigs were randomized to receive a colloid (Voluven®,HES, n=15) or crystalloid (Ringerfundin®,RF, n=15) infusion. Animals were bled till baseline SVI (Tbsl) dropped by 50% (T0), followed by resuscitation until initial SVI was reached (T4) in four steps. Invasive hemodynamic measurements, blood gas analyses and laboratory tests were performed at each assessment points. Glycocalyx degradation markers (Syndecan-1/hematocrit ratio, Glypican/hematocrit ratio) were determined at Tbsl, T0 and T4.

**Results:**

Similar amounts of blood were shed in both groups (HES group: 506±159 mls blood, RF group: 470±127 mls blood). Hemodynamic changes followed the same pattern without significant difference between the groups. Animals received significantly less resuscitation fluid in the HES compared to the RF-group: 425 [320-665], vs 1390 [884-1585] mls, *p* <0.001. The volume replacement ratio was 0.92 [0.79-1.54] for HES; and 3.03 [2.00-4.23] for the RF-group (*p* <0.001). There was no significant difference between the groups in the glycocalyx degradation markers.

**Conclusion:**

In this moderate bleeding-resuscitation animal model the volume-replacement ratio for crystalloids and colloids followed similar patterns as predicted by Starling's principle, and the glycocalyx remained intact. This indicates that in acute bleeding events, such as trauma or during surgery, colloids may be beneficial as hemodynamic stability may be achieved more rapidly than with crystalloids.

## Background

Acute bleeding is a perilous condition requiring immediate intervention before hypoperfusion leads to severe organ damage and multiple organ dysfunction. In addition to the surgical control of bleeding, fluid resuscitation remains one of the most important life-saving interventions. The use of colloids and crystalloids for resuscitation of bleeding patients has previously remained controversial with no definitive answer for the best course of action [1–4]. In trauma patients, hemorrhage has been proposed as the second most common contributing cause of death within 48 h following the injury [5, 6]. In a multi-center analysis by Hoyt et al., hemorrhage was the primary cause of intraoperative death in 82% of patients with major trauma [7]. To avoid the lethal consequences of severe bleeding, intravenous fluid resuscitation is the first line of treatment, which has to be fast and efficient.

Fundamentally, crystalloids or colloids can be used for this purpose. However, ever since colloids appeared on the scene, debate over their efficacy and potential advantages over crystalloids has continued. According to Starling’s “three-compartment model,” crystalloids, with their sodium content similar to that of the serum, are distributed in the extracellular space, while colloids should remain intravascularly due to their large molecular weight. Therefore, theoretically four times more crystalloids should have the same volume expanding effect as colloids [8]. However, crystalloid overload can also have detrimental effects; therefore, using the right kind of fluid in appropriate amounts at the right time might improve patient outcome [9].

Nevertheless, several studies including thousands of critically ill patients have seemingly disapproved the Starling principle [10–15], concluding that there were only marginal differences in the administered volume of crystalloid and colloid solutions. However, these results might have been affected by the fact that most of the included patients were septic in whom the endothelial glycocalyx layer is often found to be impaired or destroyed, resulting in increased capillary permeability. Hence, colloids may disappear into the interstitial space in larger volumes than when the glycolcalyx is intact [16, 17]. Furthermore, as reported in recent prospective studies [18, 19], non-survivor trauma patients also had significantly higher circulating syndecan-1 concentrations than survivors, indicating an impairment in the endothelial glycocalyx [16, 20, 21]. These results suggest that critical illness in general predisposes the patient to glycocalyx damage; hence, the volume-replacement ratio of crystalloids and colloids may be different from what would have been expected.

Therefore, the main aim of the current study was to compare the volume-replacement effects of crystalloid and colloid solutions during bleeding-resuscitation with moderate hemorrhage in an experimental animal model.

## Methods

The experiments were performed on the EU Directive 2010/63/EU for the protection of animals used for experimental and other scientific purposes and carried out in strict adherence to the NIH guidelines for the use of experimental animals. The experimental project was approved by the National Scientific Ethical Committee on Animal Experimentation (National Competent Authority), Hungary, with license number: V./142/2013. The study was conducted in the research laboratory of the Institute of Surgical Research in a manner that did not inflict unnecessary pain or discomfort upon the animals.

### Animals and instrumentation

Vietnamese pot-bellied pigs (*n* = 30) underwent a 12-h preoperative fasting period with free access to water. The pigs were randomized into two groups: balanced crystalloid Ringerfundin, RF group (B. Braun AG) and a colloid (Voluven®, hydroxyethyl starch (HES)) group. Anesthesia was induced by intramuscular injection of a mixture of ketamine (20 mg/kg) and xylazine (2 mg/kg), maintained by a continuous intravenous propofol infusion (6 mg/kg/h i.v.), and analgesia was performed with nalbuphine (0.1 mg/kg). Tracheal tubes were inserted in all animals, and the lungs were mechanically ventilated by Dräger Evita XL (Dräger, Lübeck, Germany). Tidal volume was adjusted to 10 mL/kg, and the respiratory rate was initialized to keep the end-tidal carbon dioxide and partial pressure of arterial carbon dioxide within physiological range (35–45 mmHg). The adequacy of anesthesia was assessed by checking jaw stiffness. After induction of anesthesia, catheters were inserted into the right jugular vein, the left carotid artery, and the right femoral artery via aseptic dissection of the vessels. For invasive hemodynamic monitoring, a transpulmonary thermodilution catheter (PiCCO, PULSION Medical Systems SE, Munich, Germany) was placed in the right femoral artery (3 mm). A central venous catheter was implanted into the right jugular vein and was positioned by the guidance of intracavital ECG. Throughout bleeding, blood was drained through a catheter from the left carotid artery to a cylinder. An external warming device was used to retain the animals’ body temperature at 37 ± 1 °C.

### Experimental protocol

**Fig. 1:**
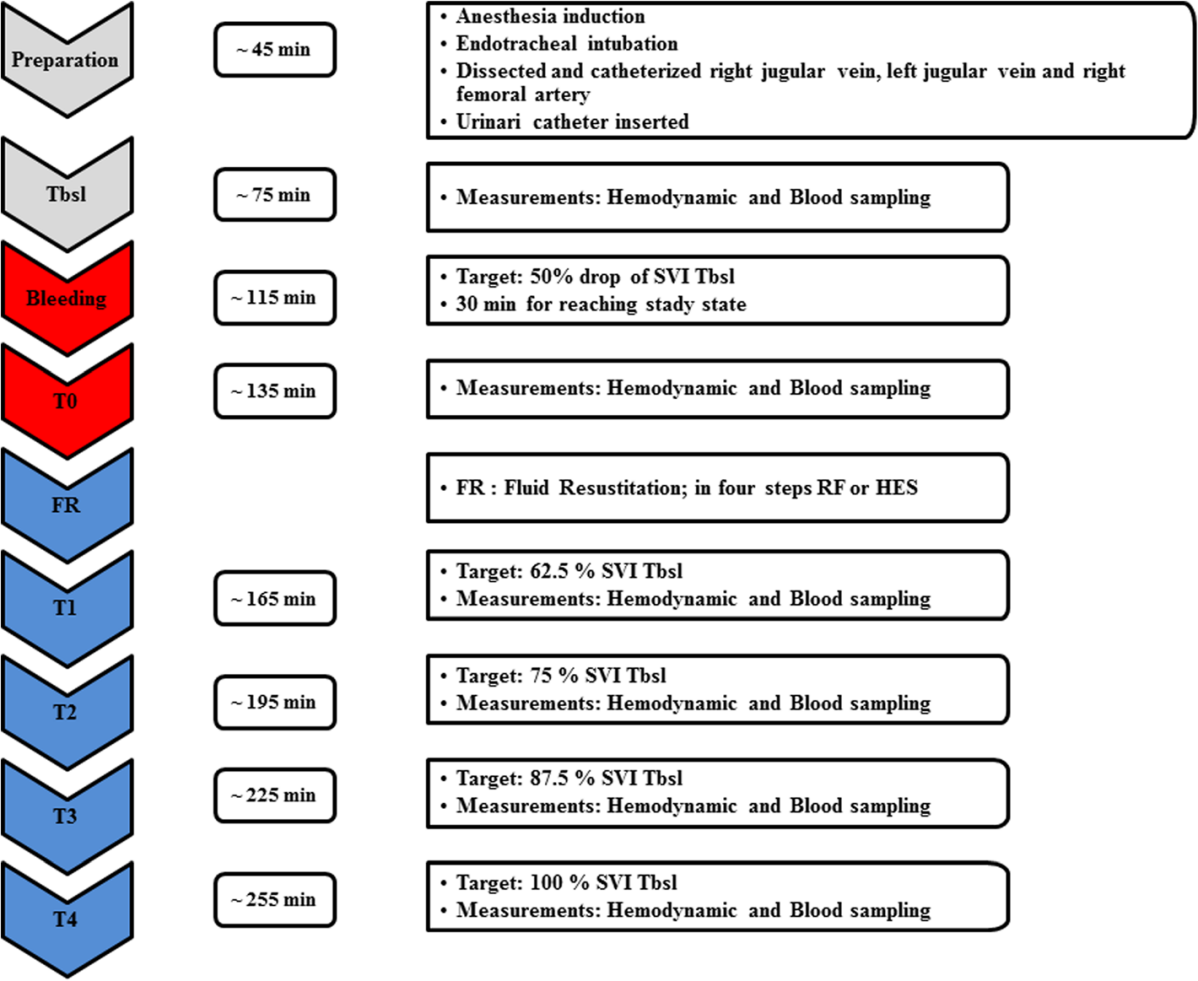
Schematic flowchart illustrating the experimental protocol. After baseline measurements, animals were bled until the stroke volume index (SVI) decreased by 50% (*T*_0_). Then, measurements were repeated and the animals were randomized into the balanced crystalloid (Ringerfundin®, RF B. Braun AG) or colloid (Voluven®, HES) groups. The difference of the SVI *T*_bsl_ − SVI *T*_0_ was divided into four equal steps (*T*_1–4_) and i.v. fluids were administered to reach these target values

We applied a model which has been tested and reported in our previous experiments [22, 23]. The study protocol is summarized in Fig. 1. Briefly, after instrumentation, 30 min was allowed for stabilization before baseline (*T*_bsl_) measurements were taken. At each assessment point, hemodynamic measurements, blood gas analyses, and laboratory tests were performed. After *T*_bsl_, the pigs were bled until the stroke volume index dropped to 50% of its baseline value (*T*_0_); then, measurements were repeated.

The difference of stroke volume index (SVI) at *T*_bsl_ and *T*_0_ was divided into four equal target values, which was planned to be reached in four steps during fluid resuscitation (*T*_1–4_) to reach the initial SVI by *T*_4_. Fluid replacement was executed with boluses of balanced RF or HES solutions until the target SVI value was reached. After reaching each step, 20 min was allowed for equilibrium; then, blood gas and hemodynamic parameters were measured. All of the pigs were euthanized with sodium pentobarbital at the end of the experiment.

### Hemodynamic monitoring and blood gas sampling

Cardiac function (CFI), cardiac index (CI), left ventricular contractility (dPmax), global end-diastolic volume (GEDI), heart rate (HR), mean arterial pressure (MAP), pulse pressure variation (PPV), stroke volume index (SVI), and stroke volume variation (SVV) were measured via transpulmonary thermodilution and pulse contour analysis at baseline and at the end of each step. All hemodynamic parameters were indexed for body surface area or body weight. Ten milliliters of less than 8 °C cold isotonic saline was injected through the jugular catheter for thermodilution-based measurements, and the average of three boluses recorded at the end of each interval. Central venous pressure (CVP) was measured via the jugular catheter in parallel with the other hemodynamic parameters. For blood gas measurements, the right femoral artery served as the site for arterial blood gas sampling and the catheter in the internal jugular vein was used for taking central venous blood gas samples. These were analyzed in parallel by co-oximetry (Cobas b 221, Roche Ltd., Basel, Switzerland) at baseline and at the end of each resuscitation step. From these parameters, the following variables were calculated [24]:

$$ \mathrm{Oxygen}\  \mathrm{consumption}\ \left({\mathrm{VO}}_2\right) $$

$$ {\mathrm{VO}}_2=\mathrm{CI}\times \left({\mathrm{CaO}}_2-\left(\mathrm{Hb}\times 1.34\times {\mathrm{ScvO}}_2+0.003\times {\mathrm{PcvO}}_2\right)\right) $$

$$ \mathrm{Oxygen}\  \mathrm{delivery}\ \left({\mathrm{DO}}_2\right) $$

$$ {\mathrm{DO}}_2=\mathrm{CI}\times \left(\mathrm{Hb}\times 1.34\times \mathrm{SaO}2+0.003\times \mathrm{PaO}2\right) $$

$$ \mathrm{Oxygen}\  \mathrm{extraction}={\mathrm{VO}}_2/{\mathrm{DO}}_2 $$

Volume-replacement ratios were calculated by the resuscitation fluid over the total blood loss.

### Glycocalyx degradation

Blood concentrations of syndecan-1 and glypican were quantified by enzyme-linked immunosorbent assay (ELISA) (MybioSource, Inc., San Diego, USA). For this purpose, blood samples were taken at *T*_bsl_, *T*_0_, and *T*_4_; then, the blood was centrifuged and the serum stored at − 80 °C.

### Data analysis and statistics

For statistical analysis, Statistical Program for Social Sciences version 23.0 for Windows (SPSS, Chicago, IL, USA) was used, and *p* < 0.05 was considered significant. Data are presented as mean ± standard deviations or median and interquartile range (IQR), respectively. For testing normal distribution, the Kolmogorov–Smirnov test was used. Independent samples were tested by independent sample *T* test or Mann–Whitney *U* test, as appropriate. Changes in repeated measures throughout the experiment were tested by two-way repeated measures analysis of variance (ANOVA) with Bonferroni post hoc comparisons. Categorical data were compared using *χ* ^2^ tests. The type I error probability associated with this test of this null hypothesis is 0.05.

## Results

Out of the 30 animals, 27 survived the full experiment. Two in the HES group and one in the RF group had a sudden cardiac arrest after induction of anesthesia for reasons unknown. Therefore, the results of 27 animals (HES *n* = 13; RF *n* = 14) were finally analyzed. Demographics and overall data on fluid management are summarized in Table 1. Animals were of similar weight, height, and body surface area in both groups. For a 50% decrease in SVI, a similar amount of blood had to be drained in both groups. Invasive hemodynamic (PiCCO) measurements were taken at similar frequencies in both groups. Urine output was significantly higher in the RF group.

**Table 1:** Demographics, blood loss, and fluid therapy

	HES (*n* = 13)	RF (*n* = 14)	*p*
Weight (kg)	26.0 [22.5–28.0]	25.5 [24.0–37.0]	0.280
Height (cm)	118.0 [112.5–120.0]	115.0 [110.0–125.0]	0.981
BSA (m^2^)	0.91[0.855–0.97]	0.94 [0.975–1.115]	0.401
Shed blood (mL)	505.6 ± 159.3	469.7 ± 127.3	0.529
Total blood loss (mL/m^2^)	552.8 ± 174.9	481.1 ± 95.2	0.197
PiCCO measurements (*n*)	23 ± 8	25 ± 5	0.422
Saline used for PiCCO measurements (mL)	230.0 ± 81.5	252.1 ± 58.2	0.422
Urine (mL)	450[350–626]^#^	759.5[421–1110]	< 0.001

Data are presented as mean ± standard deviation or median [IQR]

^#^ *p* < 0.05 significantly different between groups

### Macro-hemodynamic effects of fluid resuscitation

Hemodynamic results were similar at *T*_bsl_, and goals of 50% reduction in SVI were reached by *T*_0_ in both groups (Table 2). Hemodynamic changes during the experiment did not show clinically relevant differences between the groups. At *T*_bsl_, the SVI values were similar, after bleeding SVI decreased by the planned 50% to *T*_0_ and returned to its initial value by *T*_4_.

**Table 2:** Hemodynamic parameters during hemorrhage and fluid resuscitation

	Group	*T*_bsl_	*T*_0_	*T*_1_	*T*_2_	*T*_3_	*T*_4_
Stroke volume index (mL/m^2^)	HES	34.4 ± 7.5^†^	16.5 ± 3.6*	22 ± 4.7*^†^	25.8 ± 5.2*^†^	30.3 ± 5.7*^†^	34.3 ± 7.2^†^
	RF	33 ± 3.6^†^	15.4 ± 2.2*	19.6 ± 3.8*^†^	23.9 ± 4.0*^†^	28.0 ± 5.5*^†^	32.3 ± 3.3^†^
Cardiac index (L/min/m^2^)	HES	3.25 ± 0.23^†^	1.58 ± 0.27*	2.38 ± 0.27*^†^	2.75 ± 0.29*^†^	3.36 ± 0.34^#†^	3.99 ± 0.54^#†^
	RF	3.14 ± 0.19^†^	1.84 ± 0.4*	2.22 ± 0.43*^†^	2.52 ± 0.34*^†^	2.90 ± 0.29*^†^	3.39 ± 0.36^†^
Mean arterial pressure (mmHg)	HES	122 ± 15.2^†^	82 ± 25.9*	110 ± 27.5^#†^	114 ± 24.5^#†^	121 ± 25.2^#†^	123 ± 23.7^#†^
	RF	124 ± 16.6^†^	69 ± 17.1*	77 ± 18.3*^†^	90 ± 15.4*^†^	99 ± 18.0*^†^	101 ± 9.9*^†^
Heart rate (beats/min)	HES	95 ± 18.5	105 ± 27.5	106 ± 24.3*	106 ± 24.2*	109 ± 20.8*	117 ± 16.7_#_*^†^
	RF	97 ± 18.4^†^	111 ± 19.6*	107 ± 16.2*^†^	106 ± 18.7*^†^	102 ± 15.1^†^	102 ± 13.8^†^
Global end-diastolic volume (mL/m^2^)	HES	361 ± 60.6^†^	222 ± 36.4*	267 ± 45.2*^†^	283 ± 46.2*_†_	333 ± 54.2^#^_†_	351 ± 55.5^#^_†_
	RF	329 ± 46.8^†^	212 ± 52.5*	231 ± 51.6*^†^	249 ± 40.8*^†^	280 ± 47.3*^†^	300 ± 42.9*_†_
Stroke volume variation (%)	HES	11.4 ± 5.9^†^	23 ± 7.0*	18.1 ± 6.8*^†^	13.8 ± 3.2^†^	11.7 ± 4.7^†^	6.7 ± 2.7^#^*^†^
	RF	11.7 ± 3.0^†^	21.8 ± 6.0*	19.3 ± 5.4*	16.3 ± 4.4*^†^	13.8 ± 4.4^†^	10.3 ± 2.5^†^
Pulse pressure variation (%)	HES	10.3 ± 3.0^†^	24.2 ± 6.0*	16.6 ± 3.7^#^*^†^	13.1 ± 3.5^†^	9.8 ± 2.0^†^	6.9 ± 2.3^†^
	RF	10.5 ± 4.8^†^	24.4 ± 5.4*	22.1 ± 6.2*	16.8 ± 5.7*^†^	13.8 ± 5.6*^†^	10.3 ± 2.4^†^
Systemic vascular resistance index (dyn × s/cm^5^/m^2^)	HES	2937 ± 359	3517 ± 1094	3618 ± 831^#^*	3183 ± 650	2796 ± 483^†^	2309 ± 277*^†^
	RF	3057 ± 510	2919 ± 545	2664 ± 570	2793 ± 628	2632 ± 527*	2345 ± 433*^†^
EVLWI (mL/kg)	HES	11.22 ± 5.7^†^	10.88 ± 7.0*	11.66 ± 6.6*	12.00 ± 6.1*	12.22 ± 7.1	12.66 ± 7.0^†^
	RF	9.61 ± 2.1	9.07 ± 2.3	8.76 ± 1.2*	8.76 ± 0.9	8.84 ± 1.0	9.46 ± 1.6
dPmax (mmHg/s)	HES	703 ± 187.5^†^	612 ± 118.2*	717 ± 121.6^†^	771 ± 125.3^†^	791 ± 147.6^†^	811 ± 144.9*^†^
	RF	588 ± 246.8	588 ± 286.3	642 ± 233.0^†^	611 ± 278.3	639 ± 229.3	671 ± 223.8*^†^

Data are presented as mean ± standard deviation. HES = colloid group, RF = crystalloid group

^*^ *p* < 0.05 significantly different from *T*_bsl_

^†^ *p* < 0.05 significantly different from *T*_0_

^#^ *p* < 0.05 significantly different between groups

Kinetics of the CI, MAP, HR, and GEDI showed similar pattern in both groups with significantly higher values in the HES group at the end of the experiment (*T*_4_). SVV and PPV almost doubled after bleeding in both groups and then returned to baseline values, being significantly lower in the HES group. Extravascular lung water index showed some changes during the experiment in both groups, without any significant differences between the groups. Contractility, as indicated by dPmax values, also showed similar changes in both groups.

### Changes in VO_2_/DO_2_ during fluid resuscitation

Blood gas parameters during hemorrhage and fluid resuscitation are summarized in Table 3. Arterial pH was elevated in both groups due to unintentional hyperventilation which was then corrected towards the end of the experiment. Partial pressure of arterial oxygen tension and oxygen saturation remained stable and within the normal range throughout the study. Central venous oxygen saturation fell during the bleeding phase in both groups, but baseline values were achieved earlier in the HES group. Changes in oxygen extraction followed a similar pattern in both groups. Venous to arterial carbon dioxide gap increased significantly after the bleeding phase, with significantly higher values in the RF group, and then returned to physiological values by *T*_3_ in both groups.

**Table 3:** Blood gas parameters during hemorrhage and fluid resuscitation

	Group	*T*_bsl_	*T*_0_	*T*_1_	*T*_2_	*T*_3_	*T*_4_
pH	HES	7.613 ± 0.038^#^	7.603 ± 0.061^#^	7.568 ± 0.068^#^	7.54 ± 0.052	7.513 ± 0.043*^†^	7.519 ± 0.042*^†^
	RF	7.541 ± 0.049	7.498 ± 0.063	7.448 ± 0.055*^†^	7.393 ± 0.161*	7.464 ± 0.055*	7.48 ± 0.061*
PaCO_2_ (mmHg)	HES	28.1 ± 3.2	24.3 ± 3.8^#^	26.1 ± 4.6^#^	28.4 ± 4.4^#†^	29.9 ± 3.1^#†^	30.8 ± 4.5^†^
	RF	33.8 ± 5.3	33.9 ± 7.4	39.1 ± 6.8^†^	38.6 ± 6.8^†^	39.4 ± 6.6^†^	38.3 ± 6.0^†^
PaO_2_ (mmHg)	HES	87.5 ± 12.2^†^	102.8 ± 11.0*	100.4 ± 9.6*	95.8 ± 8.7	90.3 ± 13.0^†^	84.5 ± 14.6^†^
	RF	99.0 ± 24.7	102.0 ± 25.7	96.7 ± 27.8^†^	99.2 ± 29.3	94.9 ± 27.5	99.6 ± 30.9
HCO^−^_3_ (mmol/l)	HES	27.6 ± 2.3^†^	24.4 ± 1.8*	23.0 ± 1.5^#^*	23.1 ± 2.0*	23.5 ± 1.2^#^*	24.4 ± 1.7^#^*
	RF	28.1 ± 1.4	25.5 ± 4.0	26.3 ± 3.3	26.6 ± 3.6	27.5 ± 2.7^†^	27.7 ± 2.5^†^
SaO_2_ (%)	HES	97.8 ± 0.4	98.4 ± 0.3	98.2 ± 0.3	98 ± 0.4	97.7 ± 0.5	97.7 ± 0.4
	RF	97.8 ± 1.2	97.8 ± 1.1	96.5 ± 2.7^†^	96.9 ± 2.4	96.8 ± 2.2^†^	96.8 ± 2.6
Lactate (mmol/L)	HES	3.8 ± 2.0^†^	6.9 ± 3.7*	8.1 ± 3.0^#^*^†^	7.6 ± 2.2^#^*	7.1 ± 2.0^#^*	5.5 ± 1.9^#^*^†^
	RF	2.1 ± 0.8^†^	4.3 ± 2.0*	4.7 ± 2.0*	4.2 ± 1.8*	3.8 ± 1.7*	3.3 ± 1.4*
Hct (%)	HES	30.0 ± 4.8	26.7 ± 5.3	23.8 ± 5.2*^†^	22.5 ± 4.5*^†^	20.6 ± 3.5*^†^	19.3 ± 3.9*^†^
	RF	34.5 ± 4.6^†^	30.5 ± 5.6*	27.1 ± 4.5*^†^	25.1 ± 3.9*^†^	23 ± 2.6*^†^	22.4 ± 3.1*^†^
Hb (g/dL)	HES	9.8 ± 1.2	8.9 ± 1.4	8.1 ± 1.5*^†^	7.7 ± 1.3*^†^	7.1 ± 0.9*^†^	6.7 ± 1.0*^†^
	RF	10.9 ± 1.2	9.9 ± 1.6	9 ± 1.3*^†^	8.4 ± 1.1*^†^	7.7 ± 0.9*^†^	7.7 ± 1.0*^†^
Ca^++^ (mmol/L)	HES	0,87 ± 0,18	0,84 ± 0,23	0,84 ± 0,17	0,82 ± 0,24	0,84 ± 0,23	0,87 ± 0,29
	RF	0,93 ± 0,3	0,87 ± 0,2	0,94 ± 0,23	0,86 ± 0,25	0,91 ± 0,31	0,89 ± 0,27
K^+^ (mmol/L)	HES	2,48 ± 0,32	2,96 ± 0,49	3,04 ± 0,38*	2,98 ± 0,29*	2,9 ± 0,44	2,8 ± 0,48
	RF	2,84 ± 0,44	3,11 ± 0,33	3,33 ± 0,34*^†^	3,24 ± 0,27	3,22 ± 0,32	3,2 ± 0,27
Na^+^ (mmol/L)	HES	139,9 ± 1,2	139,5 ± 1,2	140,7 ± 0,7^†^	141,1 ± 1*^†^	141,1 ± 0,9^†^	141,2 ± 0,8^†^
	RF	138,9 ± 3,2	138,8 ± 3,2	139,7 ± 3,1^†^	139,8 ± 2,8^†^	139 ± 4,3	140,3 ± 2,9*^†^
Cl^−^ (mmol/L)	HES	98 ± 1,9	98,7 ± 2,2	99,9 ± 1,7*^†^	100,4 ± 1,3*^†^	101,1 ± 1,8*^†^	101,7 ± 1,5*^†^
	RF	97,1 ± 2,4	98,1 ± 2,7	99,6 ± 2,7*^†^	100,4 ± 2,3*^†^	100,4 ± 2,4*^†^	102 ± 2,9*^†^
Glucose (mmol/L)	HES	8,1 ± 2,2	9,3 ± 3,5	6,8 ± 2,3^†^	6 ± 1,8^†^	6,4 ± 1^†^	6,8 ± 0,8^†^
	RF	9 ± 2,9	10,9 ± 3,3	7,6 ± 3,3^†^	6,4 ± 2,9*^†^	6,2 ± 1,9*^†^	6,1 ± 1,4*
cv pH	HES	7.578 ± 0.039^#^	7.537 ± 0.07^#^	7.516 ± 0.069^#^	7.49 ± 0.047^#^*^†^	7.476 ± 0.035*^†^	7.495 ± 0.034*
	RF	7.503 ± 0.049^†^	7.416 ± 0.049*	7.39 ± 0.047*^†^	7.41 ± 0.05*	7.427 ± 0.055*	7.442 ± 0.051*
PcvCO_2_ (mmHg)	HES	31.4 ± 2.7^#^	30.3 ± 4.7^#^	30.8 ± 5.3^#^	31.7 ± 3.6^#^	33.2 ± 3.7^#^	33 ± 3.7^#^
	RF	39.4 ± 7.6	43.2 ± 7.6	47.6 ± 6.9*^†^	46.1 ± 7.1	44.3 ± 7.5	42.1 ± 6.6
PcvO_2_ (mmHg)	HES	46.7 ± 8.1^†^	36.0 ± 9.9*	40.7 ± 7.8*^†^	44.5 ± 7.8^†^	46.8 ± 5.2^†^	45.4 ± 8.7^†^
	RF	48.3 ± 7.2^†^	34.9 ± 4.0*	39.3 ± 4.1*^†^	44.8 ± 3.3^†^	46.2 ± 5.2^†^	48.6 ± 6.7^†^
ScvO_2_ (%)	HES	83.9 ± 7.3^†^	69.1 ± 14.2*	76.0 ± 9.9*	79.7 ± 5.9*^†^	82.6 ± 4.2^†^	82.8 ± 4.8^†^
	RF	83.5 ± 8.1^†^	59.9 ± 6.6*	65.7 ± 7.9*	72.7 ± 7.4*^†^	76.2 ± 6.4*^†^	79.0 ± 8.1*^†^
Oxygen delivery index (mL/min/m^2^)	HES	431.7 ± 83.9^†^	203.5 ± 48.3*	271.8 ± 55.7*^†^	295.1 ± 59.8*^†^	322.4 ± 60.7*^†^	348.1 ± 57.7*^†^
	RF	460.3 ± 43.0	251.9 ± 83.5*	269.2 ± 70.6*	282.0 ± 54.6*	296.4 ± 44.8*^†^	354.6 ± 61.6*^†^
Oxygen consumption (index mL/min/m^2^)	HES	58.6 ± 26.8	58.7 ± 27.6	62.3 ± 34.7	55.0 ± 21.8	49.7 ± 16.2	52.9 ± 20.5
	RF	65.2 ± 32.9^†^	97.5 ± 36.9*	85.3 ± 22.5*	69.4 ± 16.5^†^	63.4 ± 23.5^†^	65.5 ± 31.0^†^
Oxygen extraction (%)	HES	14.2 ± 7.2^†^	29.8 ± 14.2*	22.6 ± 9.9*	18.6 ± 6.0^†^	15.5 ± 4.5^†^	15.2 ± 5.1^†^
	RF	14.5 ± 8.2^†^	38.8 ± 6.8*	32.0 ± 7.1*	25.0 ± 6.2*^†^	21.3 ± 6.5*^†^	18.4 ± 8.3*^†^
Venous to arterial carbon dioxide gap (mmHg)	HES	3.3 ± 1.4^†^	6.0 ± 2.8^#^*	4.7 ± 2.0^#^	3.3 ± 1.9^#†^	3.3 ± 1.2^†^	2.2 ± 1.2^†^
	RF	5.6 ± 3.4^†^	9.3 ± 1.6*	8.5 ± 1.7*^†^	7.5 ± 2.6	4.9 ± 2.0^†^	3.8 ± 2.2*^†^

Data are presented as mean ± standard deviation. HES = colloid group, RF = crystalloid group

*p < 0.05 significantly different from *T*_bsl_

^†^ *p* < 0.05 significantly different from *T*_0_

^#^ *p* < 0.05 significantly different between groups

### Volume-replacement ratios

**Fig. 2:**
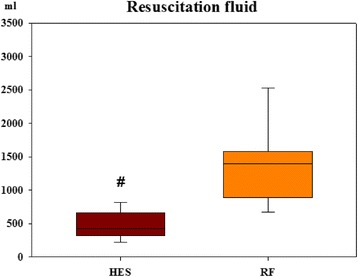
Resuscitation fluid (milliliters). Data are presented as median [IQR]. *p* = 0.002

**Fig. 3:**
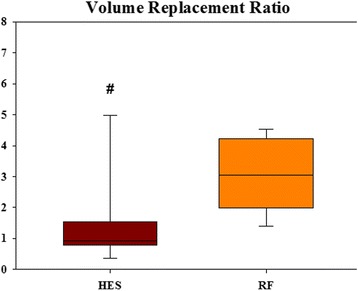
Volume-replacement ratio: resuscitation fluid/total blood loss. Data are presented as median [IQR]. *p* = 0.002

While the hemodynamic profile was very similar, there were significant differences between the groups in the total amount of fluid required and in the ratio of the resuscitation fluid over the total blood loss. Significantly more RF was used during resuscitation than HES (Fig. 2). Calculating the volume-replacement ratio, it was significantly higher in the RF group, where almost three times more RF was required to achieve the same hemodynamic parameters (Fig. 3).

### Endothelial function

**Fig. 4:**
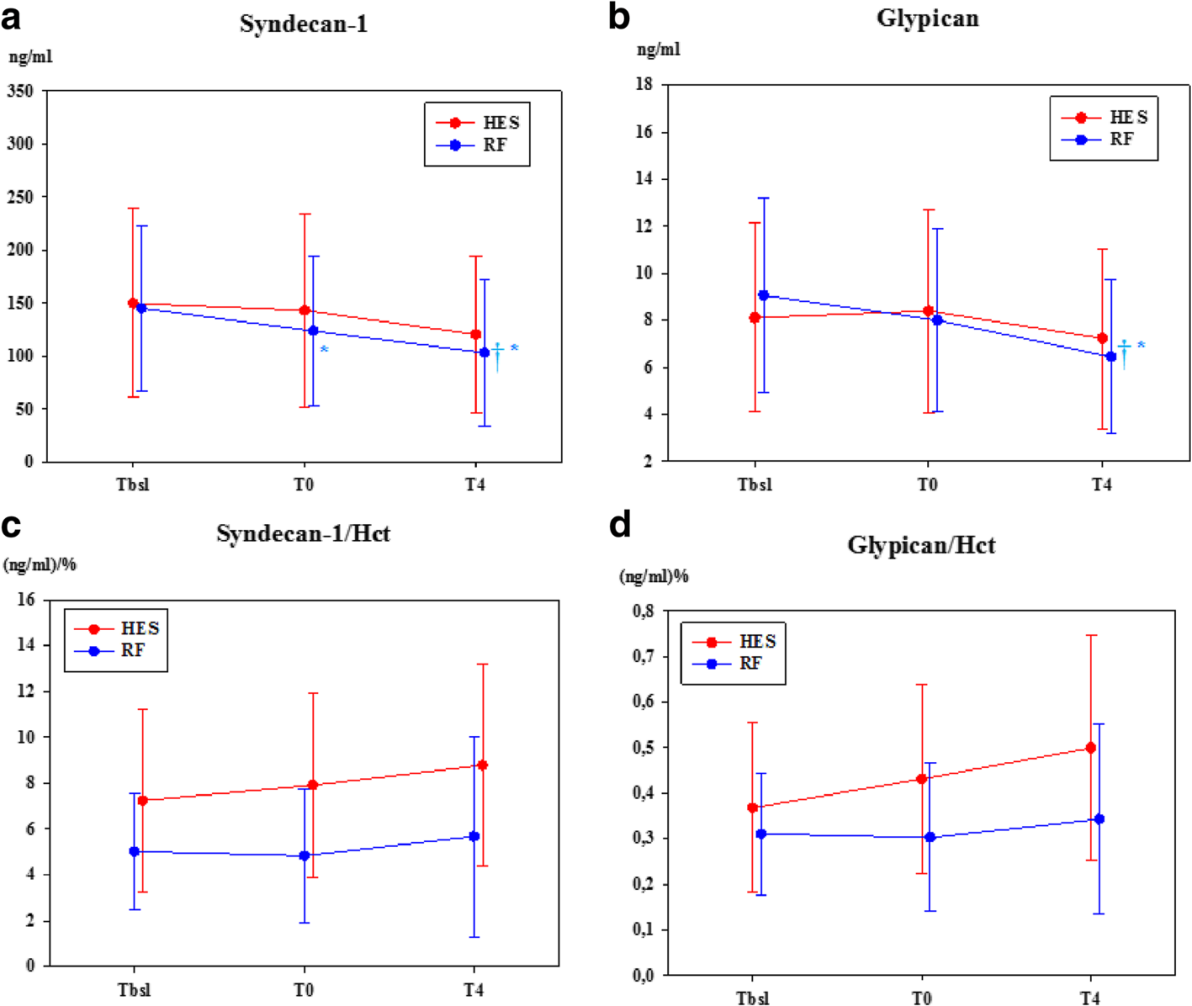
Endothelial function. Plasma concentrations of syndecan-1 (**a**), glypican (**b**), syndecan-1 hematocrit ratio (**c**) and the glypican hematocrit ratio (**d**) are delineated. Data are presented as mean ± standard deviation. HES = colloid group, RF = crystalloid group. *p < 0.05 significantly different from *T*_bsl_. ^†^ *p* < 0.05 significantly different from *T*_0_. ^#^ *p* < 0.05 significantly different between groups

Plasma concentration of syndecan-1 was significantly lower in the RF group at *T*_0_ and *T*_4_ between *T*_bsl_ values (Fig. 4a). Values of glypican in the RF group were significantly lower at *T*_4_ compared to *T*_bsl_ and *T*_0_ (Fig. 4b). However, the syndecan-1 hematocrit ratio and the glypican hematocrit ratio showed no significant differences throughout the whole experiment (Fig. 4c–d).

## Discussion

The main findings of our study are that stable hemodynamic parameters were achieved by significantly more RF than HES boluses and that the volume-replacement ratio was more than three times higher in the RF group compared to the HES group.

The results of recently performed large controlled, randomized trials on fluid therapy in the critically ill have resulted in the development of several reviews and guidelines [25–27]. Despite the vast amount of evidence on this topic, the saga of the crystalloid–colloid controversy remains an ongoing issue.

One of these landmark trials was the SAFE study, where investigators compared the safety of albumin to normal saline in ICU patients (*n* = 6997). Results showed no significant difference between the groups in the hemodynamic resuscitation end points, including mean arterial pressure or heart rate, although the use of albumin was associated with a significant but clinically small increase in central venous pressure. The study showed no significant difference between albumin and normal saline regarding 28-day mortality rate or development of new organ failure [15]. SAFE was followed by the VISEP (2008), CHEST (2012) and 6S (2012) trials [11–13]. Results showed a strong association between acute kidney injury, increased use of renal replacement therapy, and the use of hydroxyethyl starch solution, which was also accompanied with unfavorable patient outcomes. The fact that a high fraction of HES solution is deposited in the tissues [10–14] might explain the impaired organ function. On the contrary, in the Colloids Versus Crystalloids for the Resuscitation of the Critically Ill (CRISTAL) trial—which was designed to test mortality related to colloid- and crystalloid-based fluid replacement in ICU patients—investigators detected a difference in death rate after 90 days, favoring the use of colloids. Furthermore, patients spent significantly fewer days on mechanical ventilation and needed shorter durations of vasopressor therapy in the colloid group compared to the crystalloid group [10].

Regarding the volume-replacement effects, in these trials, there was a similar volume-replacement ratio for crystalloids and colloids, which is summarized in Table 4. Based on these results, a common view was formed that starch solutions do not have as high potency for volume expansion as crystalloids do, but carry a greater risk of renal dysfunction and mortality. This resulted in a dramatic decrease in synthetic colloid usage around the world.

**Table 4:** Large controlled, randomized human trials. This table summarizes large controlled, randomized human trials in terms of population, type of fluid, ratio of crystalloid (Cr)/colloid (Co) solutions, end points, outcomes, and conclusions

Trial	Population	Type of fluids	Ratio of Cr/Co	End points	Outcomes	Conclusions
Finfer 2004 SAFE (*n* = 6933)	ICU patients hypovolemic instabilities	Albumin; saline	1.32	The clinicians decided the amount and rate of fluid. The study treatment was to be used for all fluid resuscitation in the ICU until death or discharge or until 28 days following randomization.	28-day mortality, RRT, new organ failure	No significant difference in 28-day mortality rate or development of new organ failure.
Brunkhorst 2008 VISEP (*n* = 537)	Sever septic ICU patients	HES; Ringer’s lactate	1.32	CVP 8 mmHg MAP 70 mmHg ScvO_2_ 70% The treating physician decided on further measures to raise MAP and ScvO_2_ into specified range. Int he HES group: HES limit of 20 mL/kg/day	28-day mortality, RRT, new organ failure	HES was harmful, and its toxicity increased with accumulating doses.
Myburgh 2012 CHEST (*n* = 7000)	ICU patients	HES 130/0.4; Saline	1.20	Safety authority *6% HES (130/0.4) was administered to a maximum dose of 50 mL/kg/day open-label 0.9% saline for the remainder of the 24-h period	90-day mortality, AKI, ICU, and hospital stay,	No significant difference in 90-day mortality rate; however, the HES group received more RRT.
Guidet 2012 CRYSTMAS (*n* = 174)	Hemodynamic stabilization in patients with severe sepsis	HES 130/0.4; Saline	1.23	MAP ≥ 65 mmHg and at least two of the following parameters maintained for 4 h: CVP: 8–12 mmHg, urine output > 2 mL/kg, and ScvO_2_ ≥ 70% no increase in the infusion of vasopressors or inotropic therapy, and only additional study drug administration of ≤ 1 L were allowed within these 4 h	Required study fluid volume; ICU and hospital stay; SOFA score; AKI	Significantly less volume was required to achieve hemodynamic stabilization for HES vs. saline.
Perner 2012 6S (*n* = 798)	ICU patients with severe sepsis	HES 130/0.42; Ringer’s acetate	1.00	Resuscitation fluid used when ICU clinicians judged that volume expansion was needed (fluid challenge technique) CVP (8–12 mmHg); MAP (> 65 mmHg); Urine output (> 0.5 mL/kg/h); ScvO_2_ or SvO_2_(> 70%) maximum daily dose of trial fluid: 33 mL/kg of ideal body weight; maximum dose of HES: 50 mL/kg ideal BW/24 h	90-day mortality, RRT	Increased 90-day mortality with HES; increased use of RRT with HES.
Annane 2013 CRISTAL (*n* = 2857)	ICU patients with hypovolemic shock	Colloids (gelatins, dextrans, HES, 4% or 20% albumin); Crystalloids (isotonic or hypertonic saline, Ringer’s lactate)	1.5	The amount of fluid and duration of treatment was left at the investigators with restrictions: the daily maximal dose of HES 30 mL/kg of body weight investigators were required to follow any local regulatory agency recommendations governing use.	28- and 90-day mortality; days alive without the need for RRT, MV, or vasopressors	No difference in 28-day mortality; 90-day mortality lower in colloid group.
Yates 2013 (*n* = 202)	Medium- to high-risk elective colorectal surgery patients	HES 130/0.4; Hartman’s solutions	1.69	Before induction of anesthesia: 250 mL bolus of fluid was administered and the SV response recorded. If the SV increased by more than 10%, the bolus was repeated. After induction of anesthesia, further boluses of fluid were administered during surgery to maintain a SV < 10%. Volulyte up to maximum of 50 mL/kg or 5000 mL All patients received: i.v. infusion of Hartmann’s solution; rate of 1.5 mL/kg/h from the start of the trial period and this continued for 24 h. After surgery, if the urine output decreased below 0.5 mL/kg/h for 2 consecutive hours, a 250 mL bolus of fluid was administered. This continued for a 24 h period from the start of surgery.	Day 5 post-op GI morbidity; post-op complications, LOS, coagulation and inflammation	No difference in any of the measured outcomes
Caironi 2014 ALBIOS (*n* = 1810)	ICU patients with severe sepsis or septic shock	20% albumin with crystalloid; crystalloid	1.02	Albumin group: 300 mL of 20% albumin solution. From day 1 until day 28 or ICU discharge. 20% albumin was administered on a daily basis, to maintain a serum albumin level of 30 g per liter or more. *Crystalloids were administered whenever it was clinically indicated by the attending physician.	28- and 90-day mortality; organ dysfunction, LOS	No difference in mortality or other outcomes
Lobo 2010 (*n* = 10)	Healthy male subjects	Gelofusine (4% succinylated gelatine) Voluven (6% HES); saline	1.00	Compare the blood volume- expanding capacity Compare serum Na^+^, Cl^−^, HCO3^−^ concentrations Na^+^ and water excretion Plasma concentrations of hormones controlling H_2_O and Na^+^ excretion.	Changes in body water, blood volume and extra vascular fluid volume	Colloid solutions four times greater increase in blood volume as compared to saline, and extravasation was significantly higher after saline infusion.

However, it is important to note that none of these trials used detailed hemodynamic monitoring. The administration of i.v. fluids was mainly based on the clinicians’ subjective decision, or on parameters such as heart rate, blood pressure, central venous saturation, urine output, and lactate levels, none of which are a good predictor of fluid responsiveness. Linton et al. nicely showed in a postoperative critical care population that the relationship between MAP and oxygen delivery is very poor [28]. Therefore, one cannot exclude that a considerable number of these patients were not hypovolemic at all and hence received fluids unnecessarily. Nevertheless, the methods for which fluid administration was indicated in these trials, also reflects everyday practice, as was nicely shown by a large recent observational study [29]. In this survey by Cecconi et al., it was revealed that fluid therapy is mainly guided by inadequate indices. Therefore, one cannot exclude that in all the previously mentioned large trials, a considerable proportion of patients were not hypovolemic at all. This at least in part may explain the observed detrimental effects of hydroxyaethyl starches, as one cannot exclude that HES was administered to normovolemic patients; hence, its side/toxic effects were amplified. Furthermore, as it was shown in a human study with detailed blood and plasma volume assessment, different infusion volumes, infusion rates, plasma substitutes, or possibly different tracers for plasma volume measurement might lead to different results concerning the kinetics of fluid or colloid extravasation [30] .

Our intention was to perform a bleeding-resuscitation experiment with detailed hemodynamic monitoring, predefined end points, and a pragmatic protocol. During the experiment, hemodynamic changes did not show clinically relevant differences between the two groups. Kinetics of CI, SVI, MAP, HR, and GEVI showed similar patterns in both groups with significantly higher values in the HES group at the end of the experiment. The higher macro-hemodynamic values in the HES group may be due to the more rapid hemodynamic effects of colloids in general as compared to crystalloids. SVV and PPV almost doubled after bleeding in both groups and then returned to baseline values, with both being significantly lower in the HES group. Contractility, as indicated by dPmax values, also showed similar changes in both groups. In other words, we observed a similar hemodynamic course for these animals during the experiment, but the volume required was more than three times higher in the RF group. We detected elevated lactate and extravascular lung water (EVLW) levels from the start. This could have occurred during the preparation process, which may have caused some kind of distress. Nevertheless, EVLW did not reach extremely high values and regarding lactate, pigs can have higher blood lactate levels than humans, ranging from 0.5 to 5.5 mmol/L [31].

For decades, clinicians have based their choice of resuscitation fluids on Starling’s well-known compartment model. According to his principle, capillaries and post-capillary venules act as a semipermeable membrane absorbing fluid from the interstitial space [32]; hence, the hydrostatic and oncotic pressure gradients across the semipermeable membrane are the principal determinants of transvascular exchange. However, this classic model has recently been challenged [33]. One of the most important reasons why the vasculature may behave differently than that described by Starling is the recently discovered role of the glycocalyx in the function of the endothelium.

A web of membrane-bound glycoproteins and proteoglycans on the luminal side of endothelium has been identified as the glycocalyx layer. This compartment consists of many highly sulfated GAG chains providing negative charge for the endothelium. Due to these electrostatic properties, the subglycocalyx space produces a colloid oncotic pressure that might be an important determinant of vascular permeability and thus fluid balance [34]. The structure and function of the endothelial glycocalyx varies substantially among different organ systems and is affected by inflammatory conditions, such as sepsis [35]. Theoretically, with an intact glycocalyx, the volume-replacement ratio is markedly different for crystalloids compared to colloids and may behave as suggested by Starling [36]. This is also supported by other studies, including our current experiment. In a recent trial on healthy volunteers, it was demonstrated that after 1000 ml of crystalloid (isotonic saline) or colloid (gelatine and hydroxyaethyl starch) infusion, the latter caused a four times greater increase in blood volume compared to saline, and extravasation was significantly higher after saline infusion: saline 68%, gelatine 21%, and starch 16% [37]. In our experiment in healthy pigs, we also found similar differences between the volume expanding effects of RF and HES solutions. This suggests that during the early phase of bleeding, when theoretically the endothelium and the glycocalyx are intact, colloids have volume sparing effects compared to crystalloids.

### Limitations

First of all, in this model, the course of bleeding took place relatively quickly, which was almost immediately followed by resuscitation. This scenario seldom takes place during daily routine. Therefore, the results of the current study can only be partially applied to clinical practice. Another limitation is that microcirculation and extravasation of fluid was not monitored or assessed in any way; hence, the measurement of certain glycocalyx degradation molecules can only be considered as indirect indicators of glycocalyx integrity. A more detailed evaluation would be necessary to prove our concept that Starling’s theory worked in this model. Another limitation of our results is that animals remained alkalotic in the HES group as a result of unintentional hyperventilation at *T*_bsl_, *T*_0_, and *T*_1_. However, whether it interfered with the results to any extent is difficult to tell. High EVLW and lactate levels, which were elevated and remained so throughout, indicate that animal preparation, which required a considerable length of time, was not as gentle as meant to and may have caused some distress. Finally, the long-term effects of fluid resuscitation on hemodynamics, renal function, and glycocalyx degradation were not assessed; therefore, our results cannot help the crystalloid–colloid debate as far as outcomes are concerned.

## Conclusions

Our data provides experimental evidence that for the same hemodynamic effect, significantly more crystalloid than colloid solution is required in healthy pigs. Furthermore, the volume-replacement ratio was very similar to that described by Starling. Our data also suggests that the Starling’s “three-compartment model” requires an intact endothelial glycocalyx. Therefore, the clinical importance of our results is that colloids may have a place in early resuscitation before the glycocalyx suffers impairment. Further studies are required, both experimental and clinical, in which, in addition to detailed hemodynamic monitoring, the function of the microcirculation and the glycolcalyx are also assessed.
